# Volatile Organic Compound Vapour Measurements Using a Localised Surface Plasmon Resonance Optical Fibre Sensor Decorated with a Metal-Organic Framework

**DOI:** 10.3390/s21041420

**Published:** 2021-02-18

**Authors:** Chenyang He, Liangliang Liu, Sergiy Korposh, Ricardo Correia, Stephen P. Morgan

**Affiliations:** Optics and Photonics Group, Faculty of Engineering, University of Nottingham, Nottingham NG7 2RD, UK; chenyang.he2@nottingham.ac.uk (C.H.); liangliang.liu1@nottingham.ac.uk (L.L.); s.korposh@nottingham.ac.uk (S.K.); ricardo.goncalvescorreia@nottingham.ac.uk (R.C.)

**Keywords:** volatile organic compounds, metal–organic framework, localised surface plasmon resonance, optical fibre sensor

## Abstract

A tip-based fibreoptic localised surface plasmon resonance (LSPR) sensor is reported for the sensing of volatile organic compounds (VOCs). The sensor is developed by coating the tip of a multi-mode optical fibre with gold nanoparticles (size: 40 nm) via a chemisorption process and further functionalisation with the HKUST-1 metal–organic framework (MOF) via a layer-by-layer process. Sensors coated with different cycles of MOFs (40, 80 and 120) corresponding to different crystallisation processes are reported. There is no measurable response to all tested volatile organic compounds (acetone, ethanol and methanol) in the sensor with 40 coating cycles. However, sensors with 80 and 120 coating cycles show a significant redshift of resonance wavelength (up to ~9 nm) to all tested volatile organic compounds as a result of an increase in the local refractive index induced by VOC capture into the HKUST-1 thin film. Sensors gradually saturate as VOC concentration increases (up to 3.41%, 4.30% and 6.18% in acetone, ethanol and methanol measurement, respectively) and show a fully reversible response when the concentration decreases. The sensor with the thickest film exhibits slightly higher sensitivity than the sensor with a thinner film. The sensitivity of the 120-cycle-coated MOF sensor is 13.7 nm/% (R^2^ = 0.951) with a limit of detection (LoD) of 0.005% in the measurement of acetone, 15.5 nm/% (R^2^ = 0.996) with an LoD of 0.003% in the measurement of ethanol and 6.7 nm/% (R^2^ = 0.998) with an LoD of 0.011% in the measurement of methanol. The response and recovery times were calculated as 9.35 and 3.85 min for acetone; 5.35 and 2.12 min for ethanol; and 2.39 and 1.44 min for methanol. The humidity and temperature crosstalk of 120-cycle-coated MOF was measured as 0.5 ± 0.2 nm and 0.5 ± 0.1 nm in the humidity range of 50–75% relative humidity (RH) and temperature range of 20–25 °C, respectively.

## 1. Introduction

Volatile organic compounds (VOCs) are a large group of organic chemicals, including acetone, ethanol, formaldehyde and methanol, that evaporate easily at typical room temperature (~20 °C) due to high vapour pressure [1]. Emission of VOCs can be derived from a wide range of indoor and outdoor sources. Indoor sources include office supplies, household products, composite wood materials and furniture adhesives. Outdoor sources include chemical industries, food processing, vehicle manufacturers and transportation [2,3,4,5]. In fact, the majority of VOCs have inimical effects on human health, including headaches; nose, eye and throat irritation; and damage to the kidney and liver [6]. On the other hand, VOCs have the potential to be biomarkers in the applications of disease diagnosis and clinical treatments. For example, in the research relating to lung cancer, VOCs can be detected in blood samples [7], in cancerous cells directly [8,9], or in exhaled breath gas [10,11]. Breath gas analysis is a non-invasive method to identify the diseases of a patient through the detection of breath biomarkers such as VOCs, as their concentration in the breath can be related to the concentration in blood. For example, acetone, one of the breath VOCs, has been reported to present at higher levels (up to 1250 ppm) in an individual with diabetes as compared to healthy individuals (<0.76 ppm) [12,13,14]. Breath acetone is therefore considered to be one of the biomarkers for monitoring diabetes and an effective test for it could reduce the number of daily blood glucose tests in the future. Health and safety monitoring via the detection of VOC concentration in the workplace is an important action for many countries worldwide. For example, in the UK, the Health and Safety Executive (HSE) approves workplace exposure limits for acetone, ethanol and methanol of up to 1500, 1000 and 250 ppm, respectively [15]. 

The current gold standard for VOC detection uses spectrometry to conduct breath gas analysis (such as gas chromatography–mass spectrometry (GC-MS) and selected-ion flow-tube mass spectrometry (SIFT-MS)), because they provide high sensitivity and specificity [16]. However, these methods are usually time-consuming due to the extensive sample preparation required and are expensive due to the high costs of installation, method validation and personal training [17]. To satisfy the need for quick response and simple operation, one important area of research is the development of sensing devices with low-cost, fast response and portability, which could replace conventional methods. Most existing low-cost VOC sensors for workplace monitoring are electronics-based devices. However, optical fibre sensors (OFSs) inherit the geometrical advantages of an optical fibre with a diameter of approximately one hundred microns and have other benefits, such as multiplexing and immunity to electromagnetic interference. These advantages offer the possibility for a low-cost point-of-care sensor that could be used in the workplace or in healthcare clinics. The key element of chemo- and bio-optical fibre sensors is a sensitive layer that selectively traps analytes.

**Figure 1:**
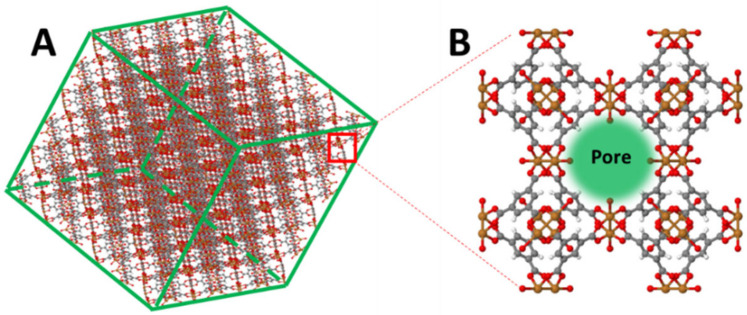
(**A**) 3D structure of HKUST-1 metal–organic frameworks. (**B**) Pore channel (green sphere) within a metal-organic framework (MOF)’s unit cell (red: oxygen, orange: metal and grey: carbon). The structural figures were modified from the ChemTube 3D website.

Metal–organic frameworks (MOFs) are constructed by linking metal-containing units with organic linkers to create open crystalline frameworks with permanent porosity [18,19]. These porous materials feature large surface area and tunable size, which are attractive for chemical sensing [20]. HKUST-1, named after the Hong Kong University of Science and Technology where it was developed, is one type of MOF that can be synthesised easily at room temperature on a functionalised surface via a layer-by-layer deposition method (LbL) [21]. This property makes it a good candidate for sensor fabrication, especially in optical-based platforms where the thickness of the film plays a vital role in sensing performance. Figure 1 illustrates the 3D structure of HKUST-1 MOFs. The porous structure, due to the presence of unsaturated copper (the diameter of the large central cavity is ~9Å), offers the possibility of capturing and concentrating gases to support highly sensitive gas sensing [22]. A study by Kim et al. demonstrated an optical sensor coated with an MOF for carbon dioxide measurement via significant intensity change [23]. However, intensity-based optical sensors are highly dependent on stable light sources and low insertion loss connectors, and are also subject to bending losses. In our previous work [24], a long-period- grating OFS with an HKUST-1 coating demonstrated a wavelength shift response to the concentration change in carbon dioxide. However, the manufacturing cost of gratings is relatively high. Moreover, when gratings are deployed in an optical fibre, they are affected by strain, which can influence the measurement of target analytes. 

Localised surface plasmon resonance (LSPR) is generated by the interaction of a light wave with the surface electrons of metallic nanoparticles (size smaller than the wavelength of light), producing localised plasmon oscillations [25]. The highly localised and intense electromagnetic field induced by LSPR renders metallic nanoparticles highly sensitive to small changes in the local refractive index with a sensitivity of about 10^−4^ refractive index units (RIUs) [26]. Due to the gold nanoparticles (Au NPs) used, the LSPR occurs at visible wavelengths, which experience wavelength shifting with refractive index change. Recently, optical fibre sensors based on LSPR have been investigated. For example, an LSPR-based U-bent plastic optical fibre sensor demonstrated a high sensitivity to refractive index change (5.57%∆A_560 nm_/RIU) in the visible region of the spectrum [27], and a reflective LSPR-based optical fibre sensor coated with silver nanoparticles demonstrated a wavelength-shifting response to refractive index change (387 nm/RIU) [28]. 

To the best of our knowledge, this work is the first demonstration of an optical fibre LSPR sensing platform with an MOF film, i.e., HKUST-1, to fabricate a new sensor. Its sensing performance was measured via sensitivity to the volatile organic compounds acetone, ethanol and methanol. Sensors with different numbers of coating cycles are reported to investigate the influence of the process of crystallisation on the sensor performance. 

## 2. Materials and Methods

### 2.1. Materials

Copper acetate (Cu_2_(AcO)_4_), 1,3,5-benzenetricarboxylic acid (H_3_(BTC)), ethanol, methanol, acetone, 11-mercaptoundecanoic acid (11-MUA), potassium hydroxide (KOH), (3-aminopropyl) triethoxysilane (APTES), trisodium citrate dihydrate, gold (III) and chloride trihydrate (HAuCl_4_·3H_2_O) were purchased from Sigma-Aldrich, UK. Acetone was purchased from Honeywell, Germany. Deionised water (DI-water), having resistivity of 18.2 Megohm-metre, was obtained from a water purification system (PURELAB Option S/R, ELGA). Pigtail multi-mode optical fibres with a core diameter of 62.5 μm (FP6ST) were obtained from All4fiber, Austria.

### 2.2. Gold Nanoparticle Synthesis 

Gold nanoparticles used in this work were synthesised via the protocol described by Turkevich et al. [29]. The 20 mM HAuCl_4_·3H_2_O stock solution was prepared in DI-water. The 90 mL DI-water and 1 mL of 20 mM HAuCl_4_·3H_2_O stock solution were mixed in a beaker and heated on a heating plate until it began to boil. Subsequently, 1 mL of sodium citrate trihydrate solution (1% *w/v* in DI-water) was added to the mixture solution. Heating continued for around 10 to 15 min until the colour of the mixture turned from transparent to red wine. Then, the gold nanoparticle solution was removed from the heating plate for cooling down to room temperature. 

### 2.3. Sensor Fabrication 

**Figure 2:**
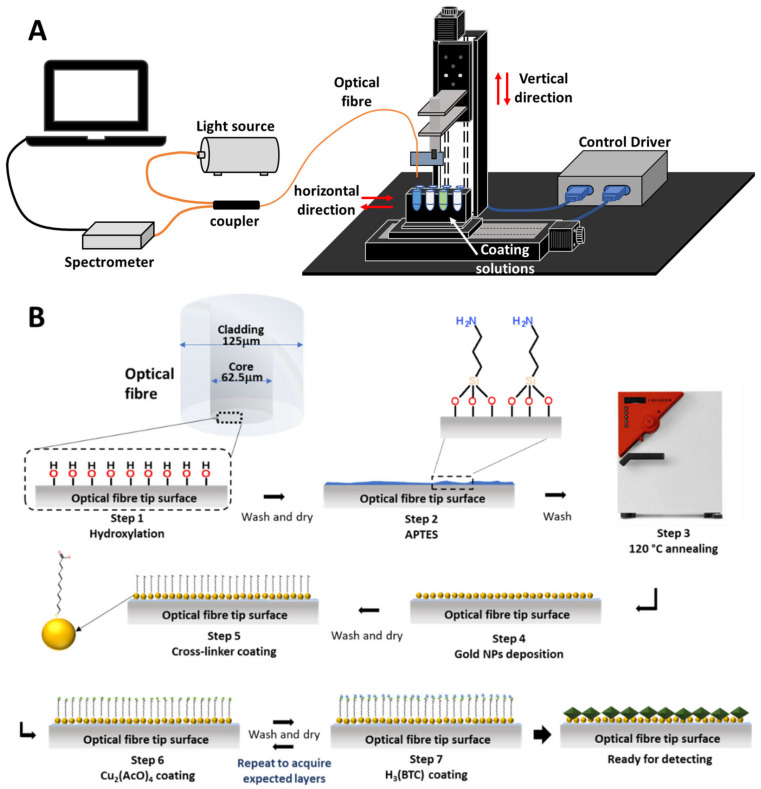
(**A**) Experimental set-up of the MOF layer-by-layer coating process. (**B**) Schematic illustration of sensor fabrication.

Figure 2 illustrates the schematic of the sensor fabrication. (1) Hydroxylation: the tip of an optical fibre was cleaved perpendicularly using a fibre cleaver (INNO, VF-78, Incheon, Korea) and hydroxylated in a 1 wt% ethanolic KOH solution (ethanol/water = 3:2. *v/v*) for an immersion time of 30 min. (2) Silanization: after drying with nitrogen, the -OH-group-linked fibre tip surface was silanized by immersing it into an APTES solution (2% *v/v* in ethanol) for 20 min to lay an amine group (-NH_2_) on the fibre surface, followed by subsequent rinsing thrice with distilled water and ethanol to remove abundant APTES from the surface. (3) LSPR functionalisation: the washed fibre was annealed for 20 min at 120 °C in an oven to remove water molecules attached on the surface and to condense the linker to the surface via the formation of siloxane bonds. Subsequently, the fibre was immersed into the prepared gold nanoparticle solution for 45 min, then washed and dried. (4) MOF functionalisation: the Au NP-coated optical fibre was further functionalised to have a carboxyl group (-COOH) terminated tail by immersing it into a 4.6 mM 11-MUA in ethanol solution for 30 min. Previous research has successfully demonstrated HKUST-1 MOF growth on carboxyl-group-terminated, self-assembled monolayers [30]. The functionalised fibre was coated with HKUST-1 MOF film by an in-house automatic deposition robot using the LbL technique, as shown in Figure 2A. In process (a), the Cu-carboxylate bond on the functionalised fibre was generated by immersing it into a negatively charged 0.2 mM Cu_2_(AcO)_4_ ethanolic solution for 5 min. In process (b), after being washed with ethanol and dried, the fibre was then immersed into a 1 mM positively charged H_3_(BTC) ethanolic solution for 5 min. The (a) and (b) processes were repeated to build the MOF on the tip surface [21]. The number of coating cycles was set to 40, 80 or 120. The growth of the film resulted from the copper acetate of the first layer binding to the –COOH group on the functionalised gold surface through a metathesis reaction to replace the acetate. In the immersion of organic ligands, the H_3_(BTC) links to the adjacent copper dimer to complete one coating cycle so that the film could be formed layer by layer. The reflection spectrum was measured using the set-up illustrated in Figure 2A, which comprises a halogen light source (HL-2000, Ocean Optics, Largo, FL, USA), a 1×2 optical fibre coupler (50%/50%, 3 dB, F-CPL-M12855, Newport, Irvine, CA, USA) and a CCD spectrometer (USB 2000, Ocean Optics, Largo, FL, USA) connected to a PC for data storage and post-processing.

### 2.4. Microscopy

The characterisation of gold NPs and HKUST-1 MOF film on the fibre tip was studied using scanning electron microscopy (SEM) (JEOL, JSM-7100F, Tokyo, Japan). The hydrodynamic size of the gold NPs was measured with dynamic light scattering (DLS, Malvern Panalytical, Malven, UK). 

### 2.5. Experimental Set-Up and Organic Vapour Measurements

**Figure 3:**
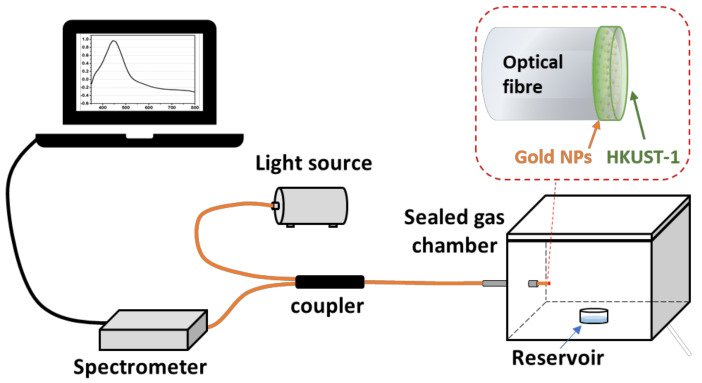
Experimental set-up of the optical fibre sensors (OFS) for sensing organic vapours. The inset figure illustrates the configuration of the coated fibre tip.

The integrated sensing system for the measurement of organic vapours (acetone, ethanol and methanol) is illustrated in Figure 3, which consists of a halogen light source (HL-2000, Ocean Optics, Largo, FL, USA), an HKUST-1 coated optical fibre probe spliced to a 2 × 1 optical fibre coupler (3 dB, F-CPL-M12855, Newport, UK), and a charge-coupled device (CCD) spectrometer (USB 2000, Ocean Optics, Largo, FL, USA) connected to a PC for data storage and post-processing. The spectrometer recorded the absorption spectrum with a sampling frequency of 1 Hz. The wavelength location of the peak absorption in the spectrum was obtained using the “findpeaks” function in MATLAB. Wavelength shifts of this “Peak Wavelength” are related to gas concentration. 

The OFS was inserted into a closed acrylic gas chamber with a total volume of 1.56 L (15 cm (L) × 13 cm (W) × 8 cm (H)). The concentration of target chemicals inside the chamber was regulated by naturally evaporating different volumes (10, 20, 40, 80, 160 µL) inside the sealed chamber. Analyte solutions of different volumes were separately added into the glass reservoir until all the liquid evaporated. The OFSs were exposed to acetone, ethanol and methanol concentrations ranging from 0.21 to 3.41%, 0.27 to 4.30% and 0.39 to 6.18%, respectively. The concentration of the analyte was calculated according to the quantity of the solution injected and the volume of the box (formula: concentration (ppm) = 24.45 × concentration (mg/m3) ÷ molecular weight [31]). All experiments were conducted in a temperature-controlled laboratory at 20.1 ± 0.1 °C. 

Cross-sensitivity to humidity and temperature was characterised by placing the MOF optical fibre sensor and a commercial capacitive temperature and humidity sensor (SparkFun BME280, Bosch, Gerlingen, Germany, relative humidity (RH) range: 0–100%RH, accuracy ±3%RH in the temperature range of −40 to 85 °C) into a climatic chamber (CVMS Climatic, Benchtop C-TH40, Harlow, UK) which can set the RH in the range from 20 to 98%RH (± 2.5%RH) and temperature from −20 to 180 °C (fluctuation: ±0.5 °C). In the humidity cross-sensitivity test, the relative humidity in the climatic chamber was controlled gradually from 50% to 75%RH, while temperature was fixed at 20 °C. Similarly, in the temperature cross-sensitivity test, the temperature was controlled gradually from 20 to 25 °C, while relative humidity level was fixed at 50%RH. 

## 3. Results and Discussion

### 3.1. Characterisations of Gold Nanoparticles and HKUST-1 Films 

**Figure 4:**
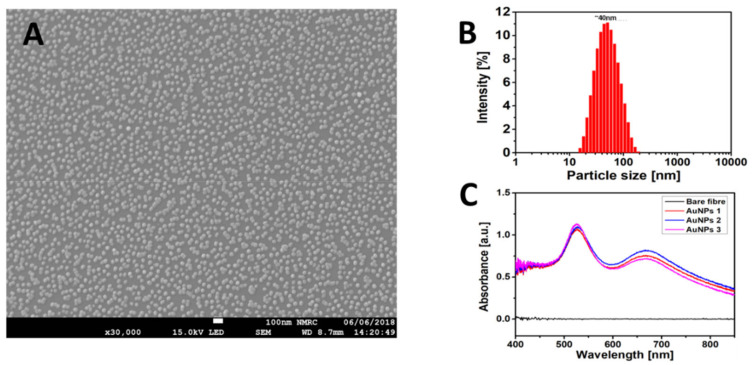
(**A**) SEM of Au NP coating on the optical fibre surface with a scale of 100 nm. (**B**) Size distribution of Au NPs obtained via dynamic light scattering (DLS). (**C**) Absorption spectra of optical fibre before and after Au NPs. Three optical fibres were coated to investigate repeatability of the coating process.

The characterisation of Au NPs is shown in Figure 4. According to the SEM illustrated in Figure 4A, the prepared Au NPs were coated successfully and uniformly on the optical fibre surface by following the fabrication protocol explained in Section 2.3. The average particle size measured using DLS was 40 nm (Figure 4B). Moreover, repeatability was verified according to the absorption spectra of optical fibre with Au NPs coating, as illustrated in Figure 4C. After coating an Au NP film on three optical fibres, absorption peaks appeared at the wavelengths of 500 and 550 nm due to the LSPR of Au NPs. The former was the plasmon resonance of individual nanoparticles, and the latter appearing at the higher wavelength was due to the overlapped plasmon distribution between two or more closely located Au NPs, as can be seen in Figure 4A [32].

**Figure 5:**
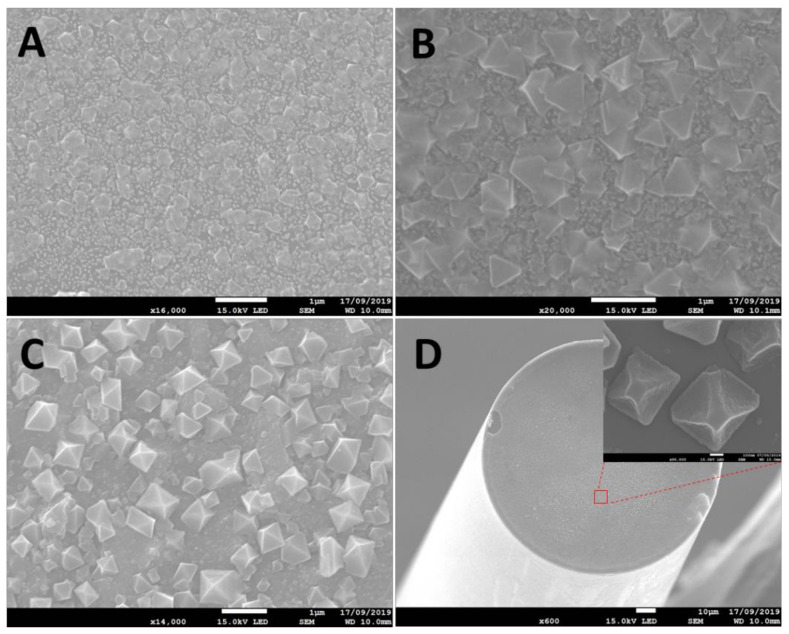
SEM images of HKUST-1 films: (**A**) 40-cycle-coated film (scale bar: 1 μm), (**B**) 80-cycle-coated film (scale bar: 1 μm), (**C**) 120-cycle-coated film (scale bar: 1 μm), (**D**) optical fibre tip coated with a film with 120 coating cycles (scale bar: 10 μm and 100 nm (inset image)).

Homogenous crystalline HKUST-1 films were successfully formed on a gold nanoparticle-coated optical fibre surface using the layer-by-layer method described in Section 2.3. SEM images (Figure 5A) show that the uniform seeds of HKUST-1 crystal were initially observed on the 40-cycle-coated MOF fibre surface, and that the crystals appear less well-formed and smaller. Subsequently, it can be clearly seen in Figure 5B that the pyramid-shaped HKUST-1 crystals gradually grew on the fibre surface, and then distinct structures were observed on the 120-cycle-coated HKUST-1 film (Figure 5C). Figure 5D illustrates the integrated optical fibre sensor tip coated with 120-cycle-coated HKUST-1 film.

**Figure 6:**
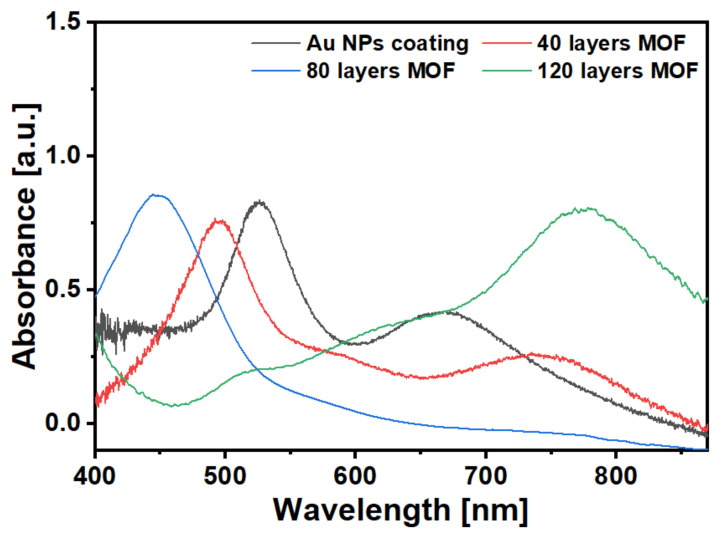
The absorption spectra of an OFS coated with Au NPs only (black trace) and Au NPs plus MOF film coated with 40 cycles (red trace), 80 cycles (blue trace) and 120 cycles (green trace). The absorbance value of the OFS is calculated by referencing the spectrum of the bare fibre.

The absorption spectra shown in Figure 6 were measured using a CCD spectrometer (USB 2000, Ocean Optics, Largo, FL, USA), which demonstrates the spectral differences of the OFS coated with HKUST-1 MOF film consisting Au NPs at 40-cycle, 80-cycles and 120-cycles of coating. The absorption spectrum peak experienced a blueshift from initial Au NP coating to 80 cycles of coating. An absorption spectrum peak appeared at ~770 nm in 120-cycle HKUST-1 MOF-coated optical fibre [33]. This peak is due to the d-d band typical of Cu(II) carboxylate complexes [34,35] and has been reported in other relevant MOF research [36,37]. 

### 3.2. Volatile Organic Compound Sensing 

**Figure 7:**
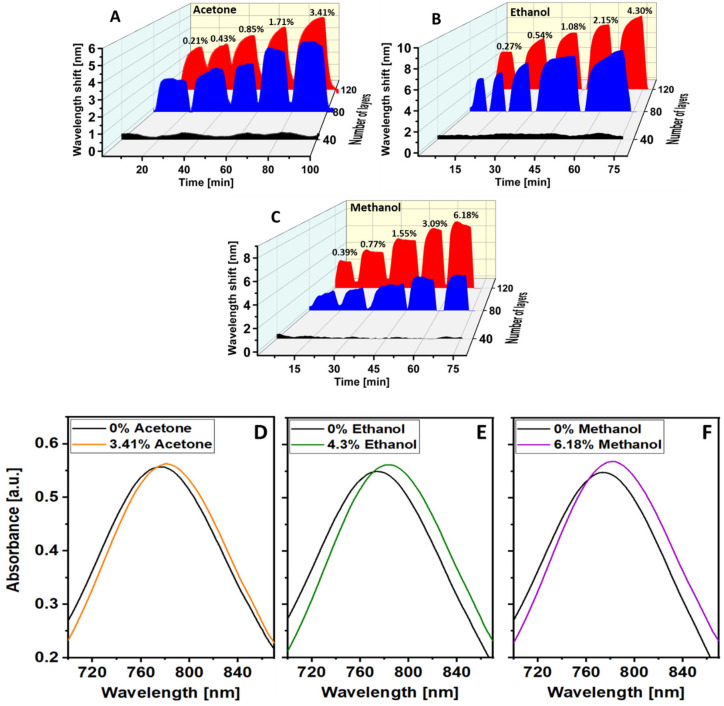
The dynamic response of peak wavelength position of the optical fibre sensor coated with 40 (black trace), 80 (blue trace) and 120 (red trace) coating cycles of HKUST-1 to different concentrations of (**A**) acetone, (**B)** ethanol and (**C**) methanol. The spectral changes of the 120-cycle-coated sensor to different concentrations of (**D**) acetone, (**E**) ethanol and (**F**) methanol.

The MOF OFSs were exposed to increasing concentrations of VOCs (acetone, ethanol and methanol) via the experimental set-up shown in Figure 3. The dynamic change of the peak wavelength to different concentrations of each VOC in a discrete exposure is shown in Figure 7. There was no obvious wavelength shift in the VOC measurements in the case of the 40-cycle-coated MOF sensor, which is illustrated as black traces in Figure 7. This was likely caused by the gas storage capacity of the 40-cycle coating not being as high as that of the thicker coatings. Compared with the 40-cycle-coated OFS, the peak wavelength responses in the 80-cycle- and 120-cycle-coated sensors (blue and red trace in Figure 7) showed a significant wavelength shift from increasing concentrations of target VOCs, as expected. Moreover, more clearly structured HKUST-1 crystals were observed in the 80-cycle and 120-cycle coating SEM images (Figure 5B,C), which should also aid in the gas-capturing ability of the film. The wavelength shift was caused by the increase in the local refractive index induced by the adsorption of VOCs inside the MOF. In addition, these dynamic results indicate that the proposed sensor also exhibits reversibility after removing the sensor back to ambient (considered as VOC-free). 

**Figure 8:**
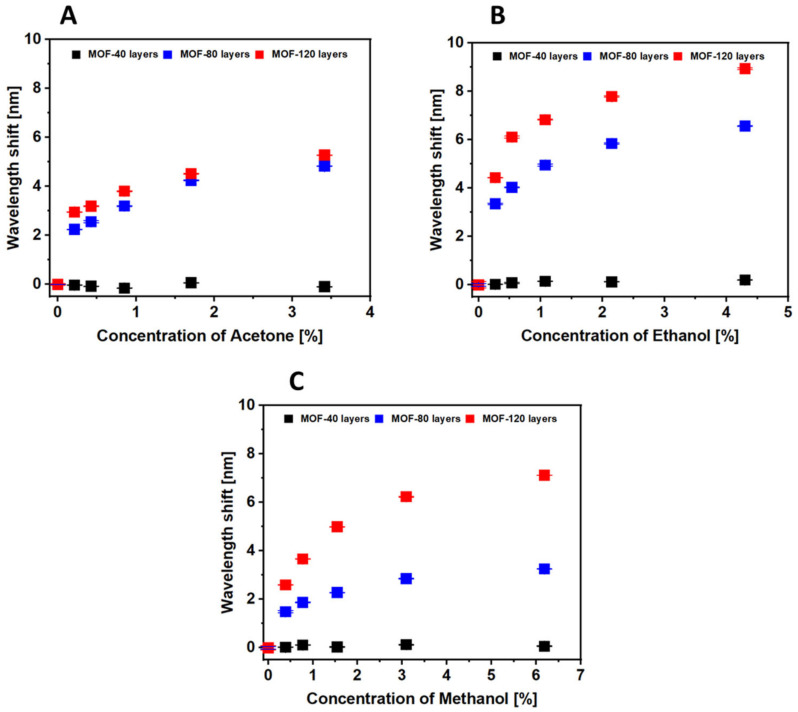
Dynamic response of the peak position of the optical fibre sensor with 40 (black trace), 80 (blue trace) and 120 coating cycles (red trace) of HKUST-1 to different concentrations of (**A**) acetone, (**B**) ethanol and (**C**) methanol.

A sensitivity comparison of 40-, 80- and 120-cycle-coated HKUST-1 sensors is depicted in Figure 8, which shows the shift of the wavelengths plotted against the concentration of the analytes. The responses were calculated by taking the average of the signal remaining at a constant level in each concentration for one minute, and the error bars are smaller than the markers. The average error bar of 120-cycle-coated sensor is 0.016, 0.026 and 0.011 nm in the acetone, ethanol and methanol measurements, respectively. Both the 80-cycle and 120-cycle-coated sensors showed a gradual saturation in the signal as the concentration of analytes increased, which may indicate that the inner volume of the HKUST-1 film was close to full. As shown in Figure 8, the response of the 120-cycle-coated sensor was higher than that of the 80-cycle-coated sensor for all VOC measurements, indicating that a sensor with well-formed HKUST-1 MOF crystals has higher sensitivity. It should be noted that the responses to VOCs of these sensors are not linearly fitted over the whole detected ranges and are more sensitive in the lower concentration range. 

**Figure 9:**
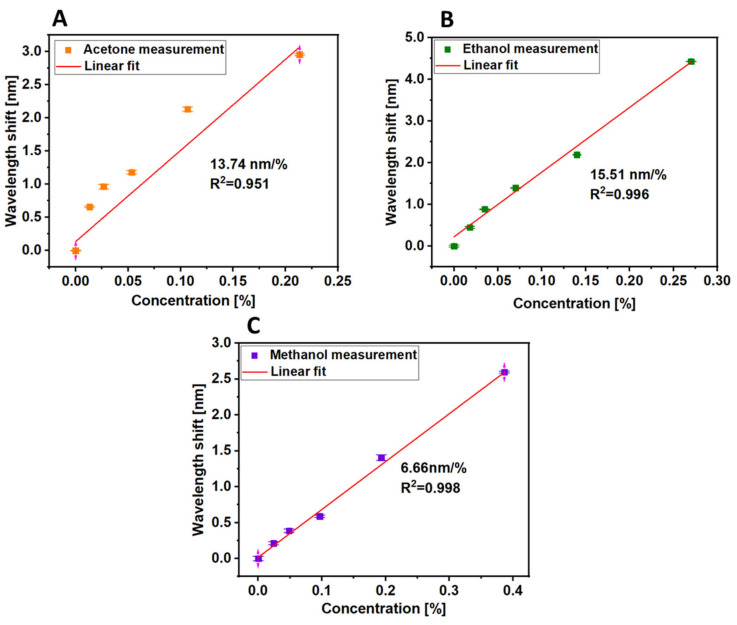
Sensitivity investigation of 120-cycle-coated MOF sensor for different VOCs: (**A**). acetone in range of 0.0–0.21% (yellow points), (**B)**. ethanol in range of 0.0–0.27% (green points) and (**C)**. methanol in range of 0.0–0.39% (purple points).

The sensitivity and the limit of detection (LoD) of VOCs of the 120-cycle-coated MOF sensor were measured. Figure 9 illustrates the response of a 120-cycle-coated MOF sensor to low concentrations of acetone, ethanol and methanol up to 0.21, 0.27 and 0.38%, respectively. The LoD is estimated as LoD = 3*σ/S, where σ is the standard deviation of the measured signal and S is the sensitivity of the sensor derived from the calibration curve [38]. Overall, the proposed sensor exhibited sensitivities to acetone, ethanol and methanol of 13.7 nm/% (R^2^ = 0.951) with LoD of 0.005% (50 ppm), 15.5 nm/% (R^2^ = 0.996) with LoD of 0.003% (30 ppm), and 6.6 nm/% (R^2^ = 0.998) with LoD of 0.011% (110 ppm), respectively.

**Figure 10:**
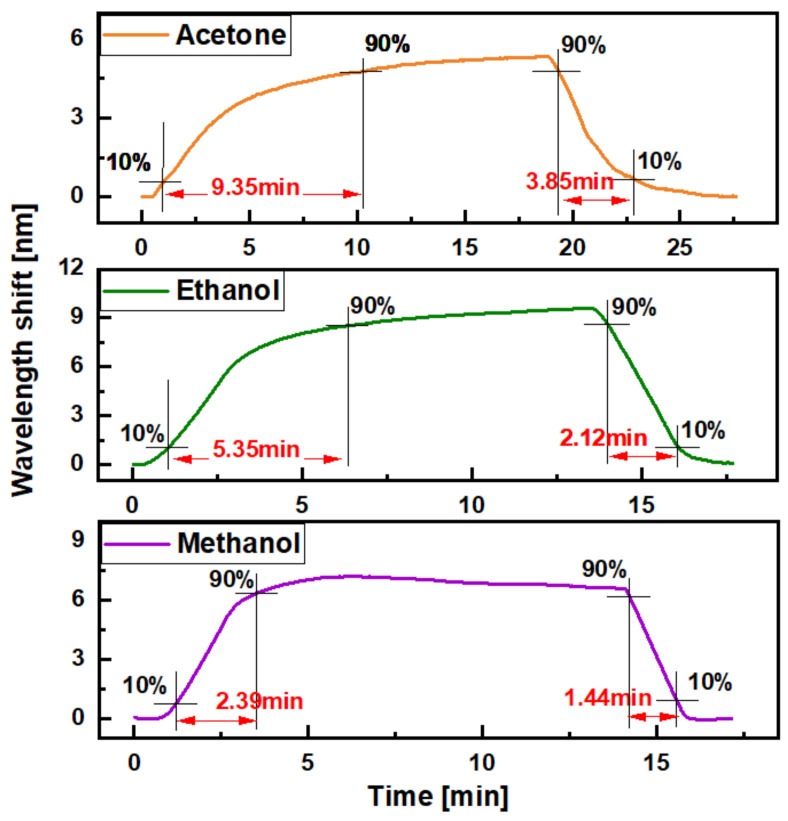
Response and recovery times of OFS for measurement of acetone (**top**), ethanol (**middle**) and methanol (**bottom**).

Figure 10 shows the response and recovery times of the 120-cycle-coated MOF sensor with acetone (orange trace on top), ethanol (green trace in middle) and methanol (purple trace on bottom) measurement, respectively. The response and recovery times are measured as the time between 10 and 90% of the signal change. In these measurements, the response and recovery time for acetone was 9.35 and 3.85 min between 0 and 3.41% concentration; the response and recovery time for ethanol was 5.35 and 2.12 min between 0 and 4.30%; and the response and recovery time for methanol was 2.39 and 1.44 min between 0 and 6.18%.

**Figure 11:**
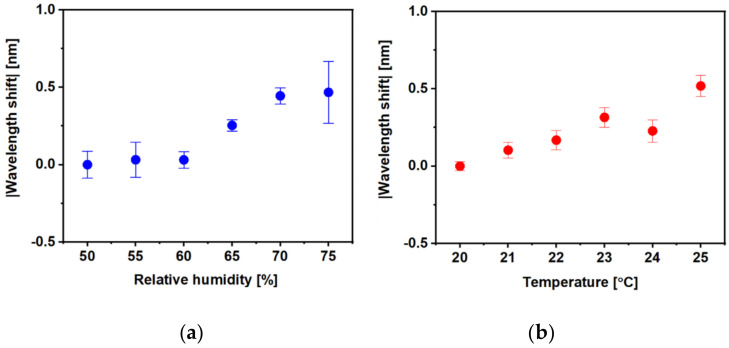
Cross-sensitivity of 120-cycle-coated MOF sensor for (**a**) humidity and (**b**) temperature.

Figure 11 illustrates the humidity and temperature crosstalk of the 120-cycle-, HKUST-1-coated sensor, in which a shift of the wavelength is plotted against a humidity change from 50 to 75%RH (Figure 11a) and a temperature change from 20 to 25 °C (Figure 11b). The sensor response to relative humidity and temperature was a small wavelength shift of 0.5 ± 0.2 and 0.5 ± 0.1 nm for a humidity change of 25%RH and temperature change of 5 °C, respectively.

Overall, the proposed MOF sensor exhibits a significant response with full reversibility to the tested VOCs. The molecular size of the VOC to be tested may affect the gas trapping process of the MOF’s pore structure on the fibre tip. For example, the methanol (32.04 g/mol) measurement had shorter response and recovery time than the measurements of acetone (58.08 g/mol) and ethanol (46.07 g/mol). As for the temperature and humidity crosstalk results, 0.5 nm wavelength shifts were recorded for 25% and 5 °C in change of relative humidity and temperature, respectively. This would be interpreted as a measurement of 0.036, 0.032 and 0.075% in acetone, ethanol and methanol, respectively. Therefore, optimised performance would be achieved in a temperature- and humidity-controllable situation and by utilising reference temperature and humidity sensors [39,40]. 

The proposed sensor has the potential to be used for workplace VOC monitoring initially, where it is not essential to discriminate between the type of VOC. However, for breath gas analysis in healthcare, the sensitivity needs to be improved to ppb levels. Sensitivity can be improved by increasing the number of coating cycles and achieving better control of the crystallinity and density of the film. Cross-sensitivity to different VOCs can be reduced by additional functionalisation of the film [41], such as through an additional selective film with specific affinity to a single VOC deposited on the topmost functional surface. A study by Farzi-kahkesh et al. developed a novel α-MoO3 hierarchical nanostructured film which presented an enhanced response to ethanol vapor [42].

## 4. Conclusions

HKUST-1 films of 40, 80 and 120 coating cycles were successfully deposited onto the surface of an optical fibre tip. The pyramid-shaped HKUST-1 crystal grows gradually with increasing numbers of coating cycles, which is confirmed by scanning electron microscopy. The reported HKUST-1 film-coated fibreoptic LSPR sensors exhibit a red spectral shift in response to increasing concentrations of volatile organic compounds (acetone, ethanol and methanol). The proposed sensors are fully reversible in the detected range from 0 to 3.41, 4.30 and 6.18% for acetone, ethanol and methanol, respectively, and are more sensitive at lower concentrations. The sensitivity of the 120-cycle-coated MOF sensor is 13.7 nm/% (R^2^ = 0.951) with an LoD of 0.005% (50 ppm) in the measurement of acetone; 15.5 nm/% (R^2^ = 0.996) with an LoD of 0.003% (30 ppm) in the measurement of ethanol; and 6.7 nm/% (R^2^ = 0.998) with an LoD of 0.011% (110 ppm) in the measurement of methanol. The response and recovery times of the 120-cycle-coated MOF sensor to acetone, ethanol and methanol were measured. Crosstalk to temperature and humidity was also investigated. This paper shows the preliminary work in the development of such sensors. The proposed sensor has the potential to be used in VOC monitoring in the workplace initially, where it is not essential to discriminate between types of VOCs. However, for breath gas analysis in healthcare, the sensitivity needs to be improved to be a ppb level, possibly by increasing the film thickness, and the cross-sensitivity needs to be reduced through an additional functionalisation of the film. 

## Data Availability

Not applicable.
